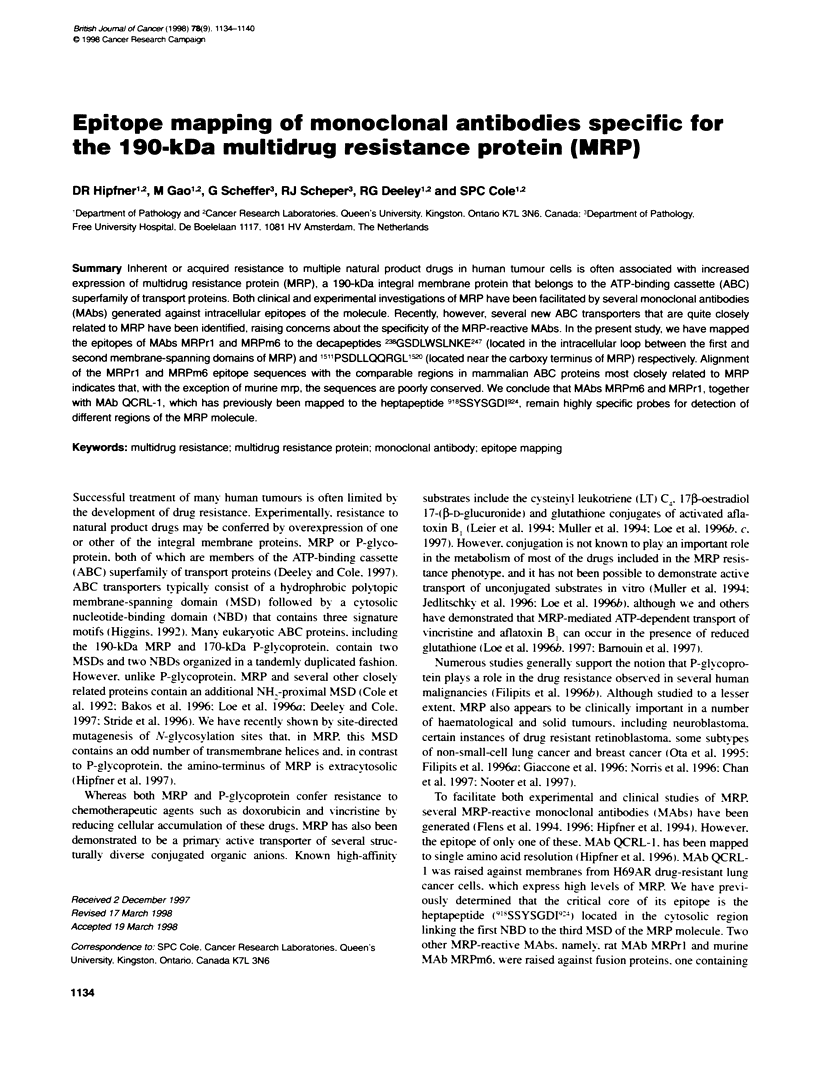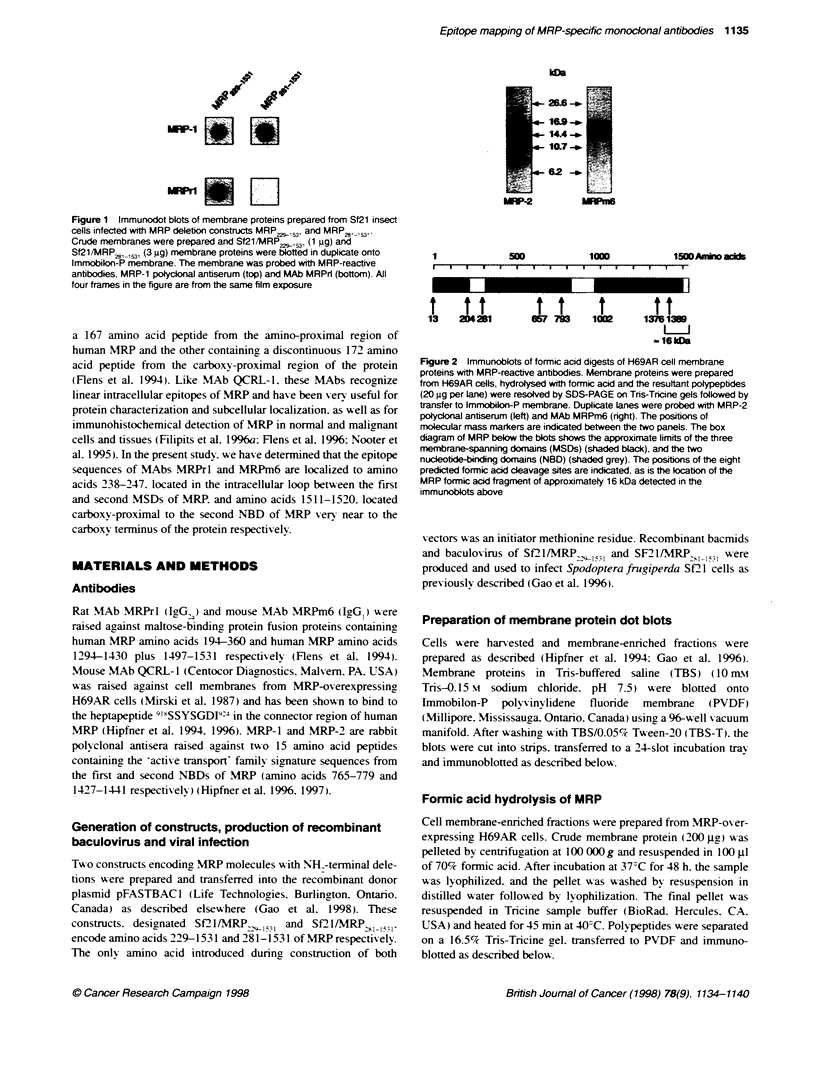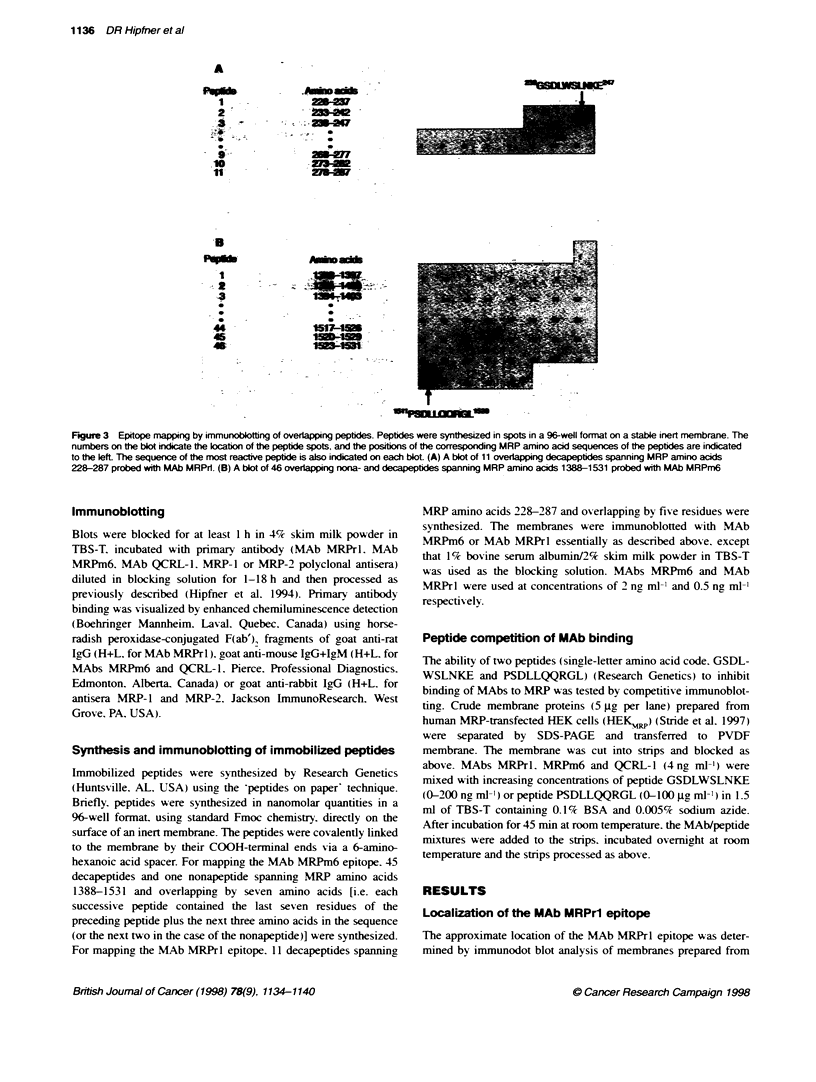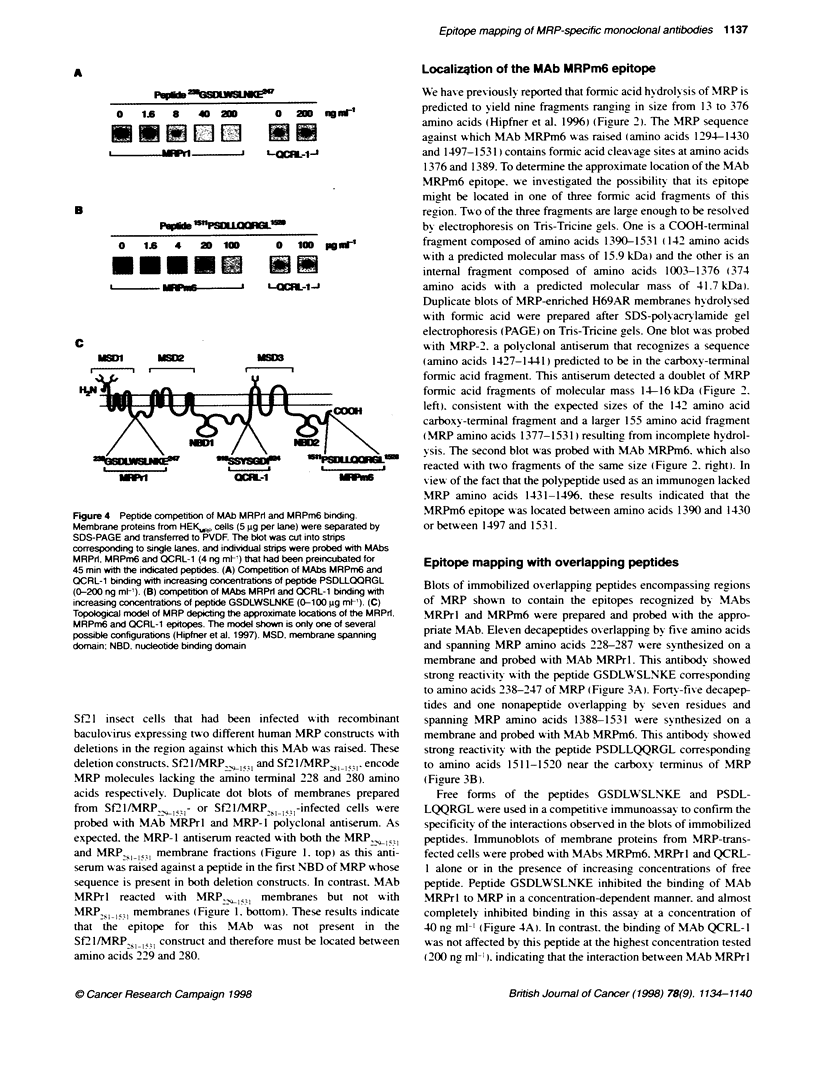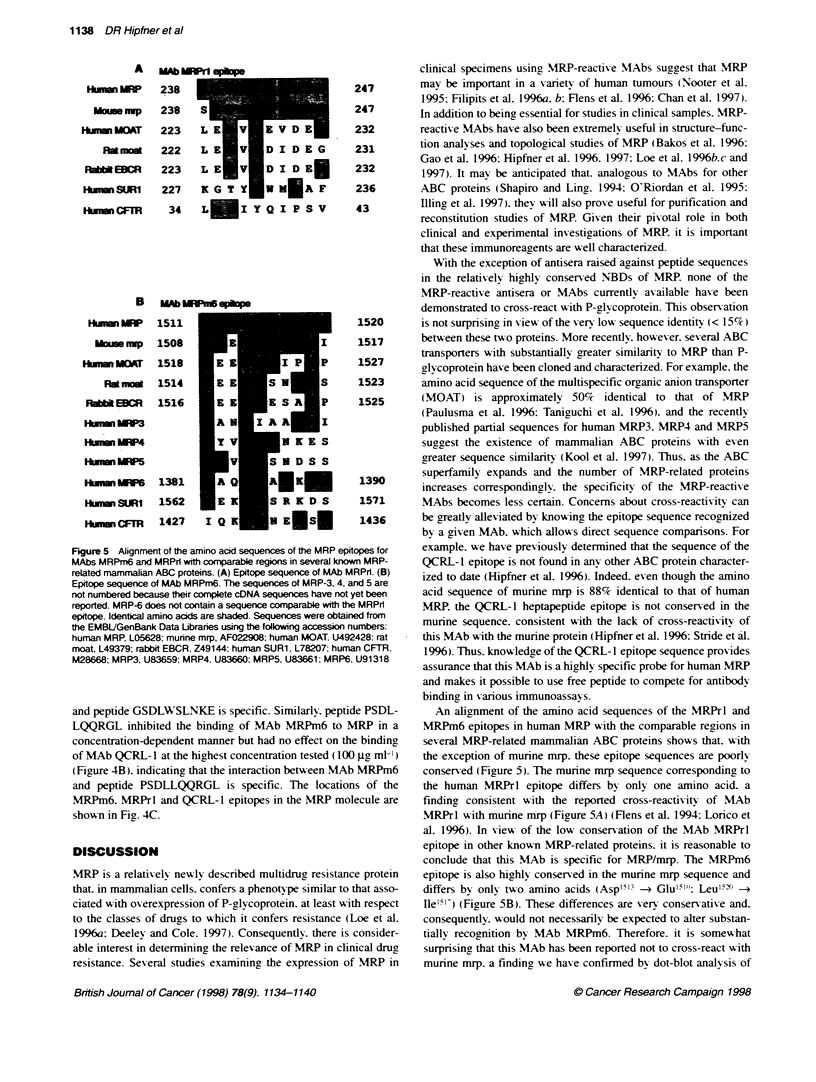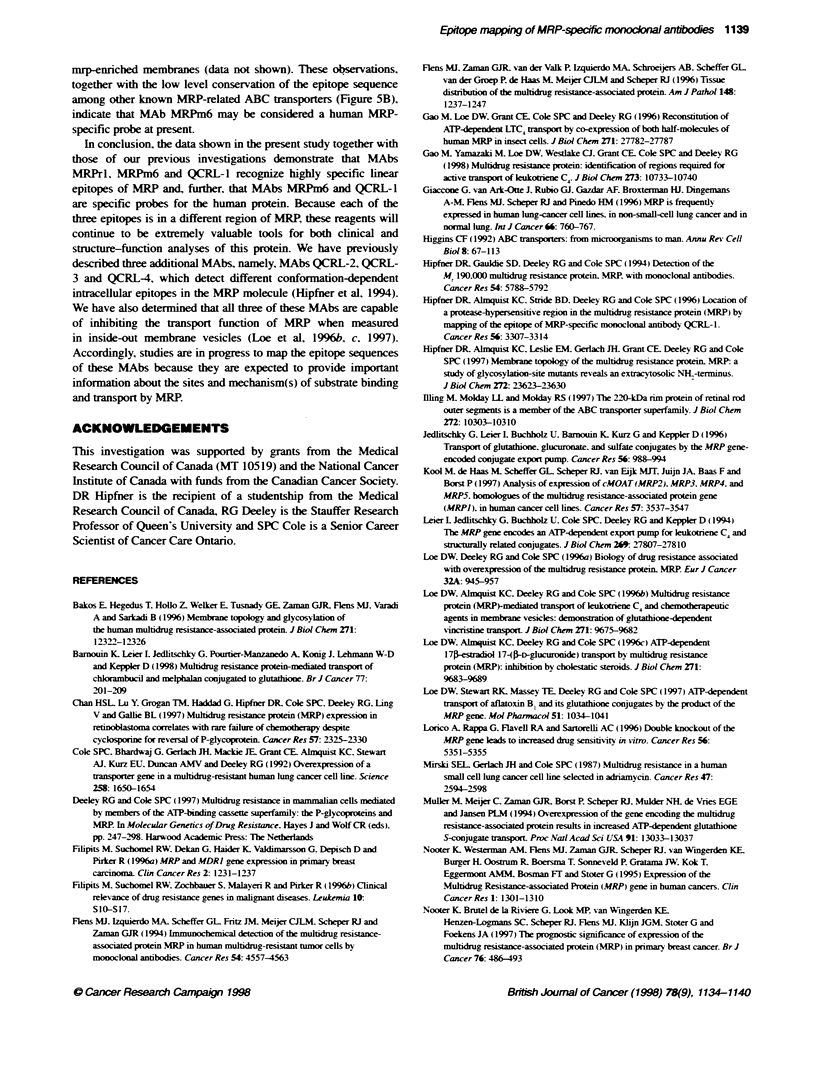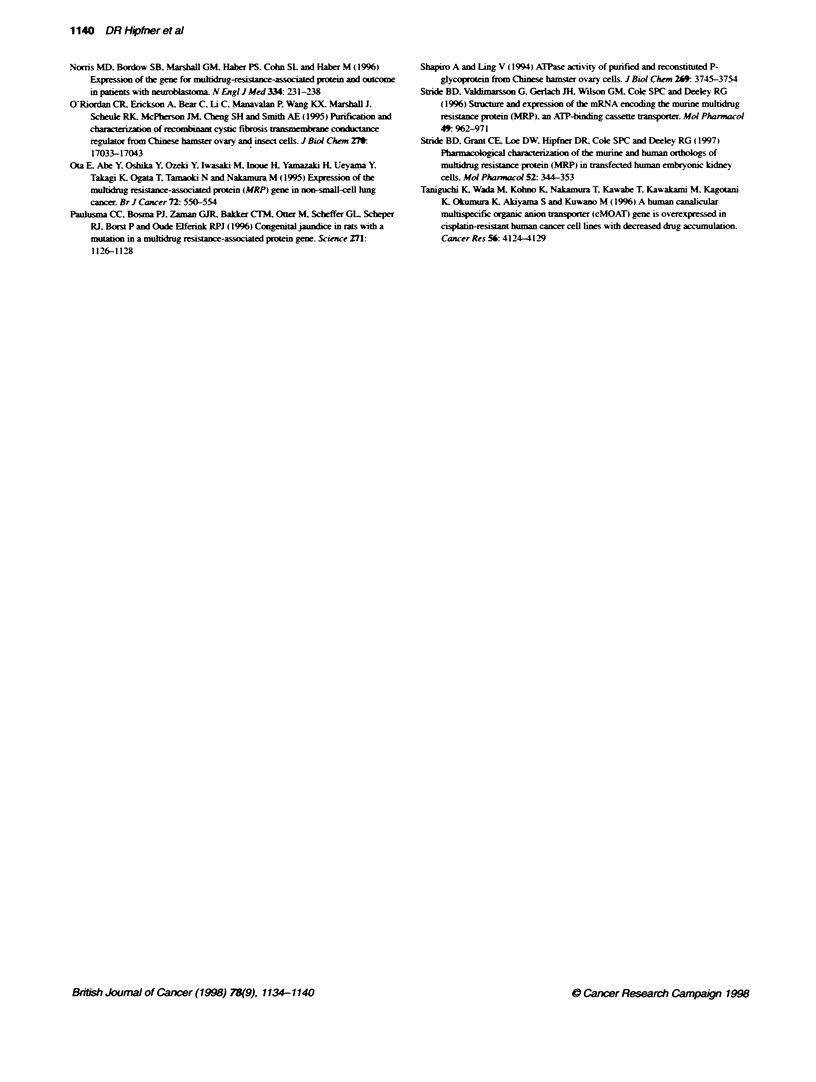# Epitope mapping of monoclonal antibodies specific for the 190-kDa multidrug resistance protein (MRP).

**DOI:** 10.1038/bjc.1998.642

**Published:** 1998-11

**Authors:** D. R. Hipfner, M. Gao, G. Scheffer, R. J. Scheper, R. G. Deeley, S. P. Cole

**Affiliations:** Department of Pathology, Queen's University, Kingston, Ontario, Canada.

## Abstract

**Images:**


British Journal of Cancer (1998) 78(9), 1134–1140
© 1998 Cancer Research Campaign

### Epitope mapping of monoclonal antibodies specific for the 190-kDa multidrug resistance protein (MRP)

**DR Hipfner[1,2], M Gao[1,2], G Scheffer[3], RJ Scheper[3], RG Deeley[1,2] and SPC Cole[1,2]**

[1]Department of Pathology and [2]Cancer Research Laboratories, Queen's University, Kingston, Ontario K7L 3N6, Canada; [3]Department of Pathology, Free University Hospital, De Boelelaan 1117, 1081 HV Amsterdam, The Netherlands

**Summary** Inherent or acquired resistance to multiple natural product drugs in human tumour cells is often associated with increased expression of multidrug resistance protein (MRP), a 190-kDa integral membrane protein that belongs to the ATP-binding cassette (ABC) superfamily of transport proteins. Both clinical and experimental investigations of MRP have been facilitated by several monoclonal antibodies (MAbs) generated against intracellular epitopes of the molecule. Recently, however, several new ABC transporters that are quite closely related to MRP have been identified, raising concerns about the specificity of the MRP-reactive MAbs. In the present study, we have mapped the epitopes of MAbs MRPr1 and MRPm6 to the decapeptides $^{238}$GSDLWSLNKE$^{247}$ (located in the intracellular loop between the first and second membrane-spanning domains of MRP) and $^{1511}$PSDLLQQRGL$^{1520}$ (located near the carboxy terminus of MRP) respectively. Alignment of the MRPr1 and MRPm6 epitope sequences with the comparable regions in mammalian ABC proteins most closely related to MRP indicates that, with the exception of murine mrp, the sequences are poorly conserved. We conclude that MAbs MRPm6 and MRPr1, together with MAb QCRL-1, which has previously been mapped to the heptapeptide $^{918}$SSYSGDI$^{924}$, remain highly specific probes for detection of different regions of the MRP molecule.

**Keywords:** multidrug resistance; multidrug resistance protein; monoclonal antibody; epitope mapping

Successful treatment of many human tumours is often limited by the development of drug resistance. Experimentally, resistance to natural product drugs may be conferred by overexpression of one or other of the integral membrane proteins, MRP or P-glycoprotein, both of which are members of the ATP-binding cassette (ABC) superfamily of transport proteins (Deeley and Cole, 1997). ABC transporters typically consist of a hydrophrobic polytopic membrane-spanning domain (MSD) followed by a cytosolic nucleotide-binding domain (NBD) that contains three signature motifs (Higgins, 1992). Many eukaryotic ABC proteins, including the 190-kDa MRP and 170-kDa P-glycoprotein, contain two MSDs and two NBDs organized in a tandemly duplicated fashion. However, unlike P-glycoprotein, MRP and several other closely related proteins contain an additional $NH_2$-proximal MSD (Cole et al, 1992; Bakos et al, 1996; Loe et al, 1996*a*; Deeley and Cole, 1997; Stride et al, 1996). We have recently shown by site-directed mutagenesis of *N*-glycosylation sites that, in MRP, this MSD contains an odd number of transmembrane helices and, in contrast to P-glycoprotein, the amino-terminus of MRP is extracytosolic (Hipfner et al, 1997).

Whereas both MRP and P-glycoprotein confer resistance to chemotherapeutic agents such as doxorubicin and vincristine by reducing cellular accumulation of these drugs, MRP has also been demonstrated to be a primary active transporter of several structurally diverse conjugated organic anions. Known high-affinity substrates include the cysteinyl leukotriene (LT) $C_4$, 17β-oestradiol 17-(β-D-glucuronide) and glutathione conjugates of activated aflatoxin $B_1$ (Leier et al, 1994; Muller et al, 1994; Loe et al, 1996*b*, *c*, 1997). However, conjugation is not known to play an important role in the metabolism of most of the drugs included in the MRP resistance phenotype, and it has not been possible to demonstrate active transport of unconjugated substrates in vitro (Muller et al, 1994; Jedlitschky et al, 1996; Loe et al, 1996*b*), although we and others have demonstrated that MRP-mediated ATP-dependent transport of vincristine and aflatoxin $B_1$ can occur in the presence of reduced glutathione (Loe et al, 1996*b*, 1997; Barnouin et al, 1997).

Numerous studies generally support the notion that P-glycoprotein plays a role in the drug resistance observed in several human malignancies (Filipits et al, 1996*b*). Although studied to a lesser extent, MRP also appears to be clinically important in a number of haematological and solid tumours, including neuroblastoma, certain instances of drug resistant retinoblastoma, some subtypes of non-small-cell lung cancer and breast cancer (Ota et al, 1995; Filipits et al, 1996*a*; Giaccone et al, 1996; Norris et al, 1996; Chan et al, 1997; Nooter et al, 1997).

To facilitate both experimental and clinical studies of MRP, several MRP-reactive monoclonal antibodies (MAbs) have been generated (Flens et al, 1994, 1996; Hipfner et al, 1994). However, the epitope of only one of these, MAb QCRL-1, has been mapped to single amino acid resolution (Hipfner et al, 1996). MAb QCRL-1 was raised against membranes from H69AR drug-resistant lung cancer cells, which express high levels of MRP. We have previously determined that the critical core of its epitope is the heptapeptide ($^{918}$SSYSGDI$^{924}$) located in the cytosolic region linking the first NBD to the third MSD of the MRP molecule. Two other MRP-reactive MAbs, namely, rat MAb MRPr1 and murine MAb MRPm6, were raised against fusion proteins, one containing

*Received 2 December 1997*
*Revised 17 March 1998*
*Accepted 19 March 1998*

*Correspondence to:* SPC Cole, Cancer Research Laboratories, Queen's University, Kingston, Ontario, Canada K7L 3N6

**Figure 1** Immunodot blots of membrane proteins prepared from Sf21 insect cells infected with MRP deletion constructs $MRP_{229-1531}$ and $MRP_{281-1531}$. Crude membranes were prepared and $Sf21/MRP_{229-1531}$ (1 μg) and $Sf21/MRP_{281-1531}$ (3 μg) membrane proteins were blotted in duplicate onto Immobilon-P membrane. The membrane was probed with MRP-reactive antibodies, MRP-1 polyclonal antiserum (top) and MAb MRPrl (bottom). All four frames in the figure are from the same film exposure a 167 amino acid peptide from the amino-proximal region of human MRP and the other containing a discontinuous 172 amino acid peptide from the carboxy-proximal region of the protein (Flens et al, 1994). Like MAb QCRL-1, these MAbs recognize linear intracellular epitopes of MRP and have been very useful for protein characterization and subcellular localization, as well as for immunohistochemical detection of MRP in normal and malignant cells and tissues (Filipits et al, 1996*a*; Flens et al, 1996; Nooter et al, 1995). In the present study, we have determined that the epitope sequences of MAbs MRPr1 and MRPm6 are localized to amino acids 238–247, located in the intracellular loop between the first and second MSDs of MRP, and amino acids 1511–1520, located carboxy-proximal to the second NBD of MRP very near to the carboxy terminus of the protein respectively.

### MATERIALS AND METHODS

### Antibodies

Rat MAb MRPr1 ($IgG_{2a}$) and mouse MAb MRPm6 ($IgG_1$) were raised against maltose-binding protein fusion proteins containing human MRP amino acids 194–360 and human MRP amino acids 1294–1430 plus 1497–1531 respectively (Flens et al, 1994). Mouse MAb QCRL-1 (Centocor Diagnostics, Malvern, PA, USA) was raised against cell membranes from MRP-overexpressing H69AR cells (Mirski et al, 1987) and has been shown to bind to the heptapeptide $^{918}$SSYSGDI$^{924}$ in the connector region of human MRP (Hipfner et al, 1994, 1996). MRP-1 and MRP-2 are rabbit polyclonal antisera raised against two 15 amino acid peptides containing the 'active transport' family signature sequences from the first and second NBDs of MRP (amino acids 765–779 and 1427–1441 respectively) (Hipfner et al, 1996, 1997).

### Generation of constructs, production of recombinant baculovirus and viral infection

Two constructs encoding MRP molecules with $NH_2$-terminal deletions were prepared and transferred into the recombinant donor plasmid pFASTBAC1 (Life Technologies, Burlington, Ontario, Canada) as described elsewhere (Gao et al, 1998). These constructs, designated $Sf21/MRP_{229-1531}$ and $Sf21/MRP_{281-1531}$, encode amino acids 229–1531 and 281–1531 of MRP respectively. The only amino acid introduced during construction of both

**Figure 2** Immunoblots of formic acid digests of H69AR cell membrane proteins with MRP-reactive antibodies. Membrane proteins were prepared from H69AR cells, hydrolysed with formic acid and the resultant polypeptides (20 μg per lane) were resolved by SDS-PAGE on Tris-Tricine gels followed by transfer to Immobilon-P membrane. Duplicate lanes were probed with MRP-2 polyclonal antiserum (left) and MAb MRPm6 (right). The positions of molecular mass markers are indicated between the two panels. The box diagram of MRP below the blots shows the approximate limits of the three membrane-spanning domains (MSDs) (shaded black), and the two nucleotide-binding domains (NBD) (shaded grey). The positions of the eight predicted formic acid cleavage sites are indicated, as is the location of the MRP formic acid fragment of approximately 16 kDa detected in the immunoblots above vectors was an initiator methionine residue. Recombinant bacmids and baculovirus of $Sf21/MRP_{229-1531}$ and $SF21/MRP_{281-1531}$ were produced and used to infect *Spodoptera frugiperda* Sf21 cells as previously described (Gao et al, 1996).

### Preparation of membrane protein dot blots

Cells were harvested and membrane-enriched fractions were prepared as described (Hipfner et al, 1994; Gao et al, 1996). Membrane proteins in Tris-buffered saline (TBS) (10 mM Tris–0.15 M sodium chloride, pH 7.5) were blotted onto Immobilon-P polyvinylidene fluoride membrane (PVDF) (Millipore, Mississauga, Ontario, Canada) using a 96-well vacuum manifold. After washing with TBS/0.05% Tween-20 (TBS-T), the blots were cut into strips, transferred to a 24-slot incubation tray and immunoblotted as described below.

### Formic acid hydrolysis of MRP

Cell membrane-enriched fractions were prepared from MRP-overexpressing H69AR cells. Crude membrane protein (200 μg) was pelleted by centrifugation at 100 000 *g* and resuspended in 100 μl of 70% formic acid. After incubation at 37°C for 48 h, the sample was lyophilized, and the pellet was washed by resuspension in distilled water followed by lyophilization. The final pellet was resuspended in Tricine sample buffer (BioRad, Hercules, CA, USA) and heated for 45 min at 40°C. Polypeptides were separated on a 16.5% Tris-Tricine gel, transferred to PVDF and immunoblotted as described below.

© *Cancer Research Campaign 1998*

**Figure 3** Epitope mapping by immunoblotting of overlapping peptides. Peptides were synthesized in spots in a 96-well format on a stable inert membrane. The numbers on the blot indicate the location of the peptide spots, and the positions of the corresponding MRP amino acid sequences of the peptides are indicated to the left. The sequence of the most reactive peptide is also indicated on each blot. (**A**) A blot of 11 overlapping decapeptides spanning MRP amino acids 228–287 probed with MAb MRPr1. (**B**) A blot of 46 overlapping nona- and decapeptides spanning MRP amino acids 1388–1531 probed with MAb MRPm6

### Immunoblotting

Blots were blocked for at least 1 h in 4% skim milk powder in TBS-T, incubated with primary antibody (MAb MRPr1, MAb MRPm6, MAb QCRL-1, MRP-1 or MRP-2 polyclonal antisera) diluted in blocking solution for 1–18 h and then processed as previously described (Hipfner et al, 1994). Primary antibody binding was visualized by enhanced chemiluminescence detection (Boehringer Mannheim, Laval, Quebec, Canada) using horseradish peroxidase-conjugated $F(ab')_2$ fragments of goat anti-rat IgG (H+L, for MAb MRPr1), goat anti-mouse IgG+IgM (H+L, for MAbs MRPm6 and QCRL-1, Pierce, Professional Diagnostics, Edmonton, Alberta, Canada) or goat anti-rabbit IgG (H+L, for antisera MRP-1 and MRP-2, Jackson ImmunoResearch, West Grove, PA, USA).

### Synthesis and immunoblotting of immobilized peptides

Immobilized peptides were synthesized by Research Genetics (Huntsville, AL, USA) using the 'peptides on paper' technique. Briefly, peptides were synthesized in nanomolar quantities in a 96-well format, using standard Fmoc chemistry, directly on the surface of an inert membrane. The peptides were covalently linked to the membrane by their COOH-terminal ends via a 6-aminohexanoic acid spacer. For mapping the MAb MRPm6 epitope, 45 decapeptides and one nonapeptide spanning MRP amino acids 1388–1531 and overlapping by seven amino acids [i.e. each successive peptide contained the last seven residues of the preceding peptide plus the next three amino acids in the sequence (or the next two in the case of the nonapeptide)] were synthesized. For mapping the MAb MRPr1 epitope, 11 decapeptides spanning MRP amino acids 228–287 and overlapping by five residues were synthesized. The membranes were immunoblotted with MAb MRPm6 or MAb MRPr1 essentially as described above, except that 1% bovine serum albumin/2% skim milk powder in TBS-T was used as the blocking solution. MAbs MRPm6 and MAb MRPr1 were used at concentrations of 2 ng $ml^{-1}$ and 0.5 ng $ml^{-1}$ respectively.

### Peptide competition of MAb binding

The ability of two peptides (single-letter amino acid code, GSDLWSLNKE and PSDLLQQRGL) (Research Genetics) to inhibit binding of MAbs to MRP was tested by competitive immunoblotting. Crude membrane proteins (5 µg per lane) prepared from human MRP-transfected HEK cells ($HEK_{MRP}$) (Stride et al, 1997) were separated by SDS-PAGE and transferred to PVDF membrane. The membrane was cut into strips and blocked as above. MAbs MRPr1, MRPm6 and QCRL-1 (4 ng $ml^{-1}$) were mixed with increasing concentrations of peptide GSDLWSLNKE (0–200 ng $ml^{-1}$) or peptide PSDLLQQRGL (0–100 µg $ml^{-1}$) in 1.5 ml of TBS-T containing 0.1% BSA and 0.005% sodium azide. After incubation for 45 min at room temperature, the MAb/peptide mixtures were added to the strips, incubated overnight at room temperature and the strips processed as above.

### RESULTS

### Localization of the MAb MRPr1 epitope

The approximate location of the MAb MRPr1 epitope was determined by immunodot blot analysis of membranes prepared from

 © Cancer Research Campaign 1998

**Figure 4** Peptide competition of MAb MRPrl and MRPm6 binding. Membrane proteins from $HEK_{MRP}$ cells (5 µg per lane) were separated by SDS-PAGE and transferred to PVDF. The blot was cut into strips corresponding to single lanes, and individual strips were probed with MAbs MRPrl, MRPm6 and QCRL-1 (4 ng $ml^{-1}$) that had been preincubated for 45 min with the indicated peptides. (**A**) Competition of MAbs MRPm6 and QCRL-1 binding with increasing concentrations of peptide PSDLLQQRGL (0–200 ng $ml^{-1}$). (**B**) competition of MAbs MRPrl and QCRL-1 binding with increasing concentrations of peptide GSDLWSLNKE (0–100 µg $ml^{-1}$). (**C**) Topological model of MRP depicting the approximate locations of the MRPrl, MRPm6 and QCRL-1 epitopes. The model shown is only one of several possible configurations (Hipfner et al. 1997). MSD, membrane spanning domain; NBD, nucleotide binding domain

Sf21 insect cells that had been infected with recombinant baculovirus expressing two different human MRP constructs with deletions in the region against which this MAb was raised. These deletion constructs, Sf21/$MRP_{229-1531}$ and Sf21/$MRP_{281-1531}$, encode MRP molecules lacking the amino terminal 228 and 280 amino acids respectively. Duplicate dot blots of membranes prepared from Sf21/$MRP_{229-1531}$- or Sf21/$MRP_{281-1531}$-infected cells were probed with MAb MRPr1 and MRP-1 polyclonal antiserum. As expected, the MRP-1 antiserum reacted with both the $MRP_{229-1531}$ and $MRP_{281-1531}$ membrane fractions (Figure 1, top) as this antiserum was raised against a peptide in the first NBD of MRP whose sequence is present in both deletion constructs. In contrast, MAb MRPr1 reacted with $MRP_{229-1531}$ membranes but not with $MRP_{281-1531}$ membranes (Figure 1, bottom). These results indicate that the epitope for this MAb was not present in the Sf21/$MRP_{281-1531}$ construct and therefore must be located between amino acids 229 and 280.

### Localization of the MAb MRPm6 epitope

We have previously reported that formic acid hydrolysis of MRP is predicted to yield nine fragments ranging in size from 13 to 376 amino acids (Hipfner et al, 1996) (Figure 2). The MRP sequence against which MAb MRPm6 was raised (amino acids 1294–1430 and 1497–1531) contains formic acid cleavage sites at amino acids 1376 and 1389. To determine the approximate location of the MAb MRPm6 epitope, we investigated the possibility that its epitope might be located in one of three formic acid fragments of this region. Two of the three fragments are large enough to be resolved by electrophoresis on Tris-Tricine gels. One is a COOH-terminal fragment composed of amino acids 1390–1531 (142 amino acids with a predicted molecular mass of 15.9 kDa) and the other is an internal fragment composed of amino acids 1003–1376 (374 amino acids with a predicted molecular mass of 41.7 kDa). Duplicate blots of MRP-enriched H69AR membranes hydrolysed with formic acid were prepared after SDS-polyacrylamide gel electrophoresis (PAGE) on Tris-Tricine gels. One blot was probed with MRP-2, a polyclonal antiserum that recognizes a sequence (amino acids 1427–1441) predicted to be in the carboxy-terminal formic acid fragment. This antiserum detected a doublet of MRP formic acid fragments of molecular mass 14–16 kDa (Figure 2, left), consistent with the expected sizes of the 142 amino acid carboxy-terminal fragment and a larger 155 amino acid fragment (MRP amino acids 1377–1531) resulting from incomplete hydrolysis. The second blot was probed with MAb MRPm6, which also reacted with two fragments of the same size (Figure 2, right). In view of the fact that the polypeptide used as an immunogen lacked MRP amino acids 1431–1496, these results indicated that the MRPm6 epitope was located between amino acids 1390 and 1430 or between 1497 and 1531.

### Epitope mapping with overlapping peptides

Blots of immobilized overlapping peptides encompassing regions of MRP shown to contain the epitopes recognized by MAbs MRPr1 and MRPm6 were prepared and probed with the appropriate MAb. Eleven decapeptides overlapping by five amino acids and spanning MRP amino acids 228–287 were synthesized on a membrane and probed with MAb MRPr1. This antibody showed strong reactivity with the peptide GSDLWSLNKE corresponding to amino acids 238–247 of MRP (Figure 3A). Forty-five decapeptides and one nonapeptide overlapping by seven residues and spanning MRP amino acids 1388–1531 were synthesized on a membrane and probed with MAb MRPm6. This antibody showed strong reactivity with the peptide PSDLLQQRGL corresponding to amino acids 1511–1520 near the carboxy terminus of MRP (Figure 3B).

Free forms of the peptides GSDLWSLNKE and PSDLLQQRGL were used in a competitive immunoassay to confirm the specificity of the interactions observed in the blots of immobilized peptides. Immunoblots of membrane proteins from MRP-transfected cells were probed with MAbs MRPm6, MRPr1 and QCRL-1 alone or in the presence of increasing concentrations of free peptide. Peptide GSDLWSLNKE inhibited the binding of MAb MRPr1 to MRP in a concentration-dependent manner, and almost completely inhibited binding in this assay at a concentration of 40 ng $ml^{-1}$ (Figure 4A). In contrast, the binding of MAb QCRL-1 was not affected by this peptide at the highest concentration tested (200 ng $ml^{-1}$), indicating that the interaction between MAb MRPr1

**A** MAb MRPr1 epitope

| | | | | | | | | | | | | |
|---|---|---|---|---|---|---|---|---|---|---|---|---|
| Human MRP | 238 | G | S | D | L | W | S | L | N | K | E | 247 |
| Mouse mrp | 238 | S | S | D | L | W | S | L | N | K | E | 247 |
| Human MOAT | 223 | L | E | D | V | W | E | V | D | E | E | 232 |
| Rat moat | 222 | L | E | D | V | W | D | I | D | E | G | 231 |
| Rabbit EBCR | 223 | L | E | D | V | W | D | I | D | E | E | 232 |
| Human SUR1 | 227 | K | G | T | Y | W | W | M | N | A | F | 236 |
| Human CFTR | 34 | L | S | D | I | Y | Q | I | P | S | V | 43 |

**B** MAb MRPm6 epitope

| | | | | | | | | | | | | |
|---|---|---|---|---|---|---|---|---|---|---|---|---|
| Human MRP | 1511 | P | S | D | L | L | Q | Q | R | G | L | 1520 |
| Mouse mrp | 1508 | P | S | E | L | L | Q | Q | R | G | I | 1517 |
| Human MOAT | 1518 | P | E | E | L | L | Q | I | P | G | P | 1527 |
| Rat moat | 1514 | P | E | E | L | L | S | N | R | G | S | 1523 |
| Rabbit EBCR | 1516 | P | E | E | L | L | E | S | A | G | P | 1525 |
| Human MRP3 | | P | A | N | L | I | A | A | R | G | I | |
| Human MRP4 | | P | Y | V | L | L | Q | N | K | E | S | |
| Human MRP5 | | P | S | V | L | L | S | N | D | S | S | |
| Human MRP6 | 1381 | P | A | Q | L | L | A | Q | K | G | L | 1390 |
| Human SUR1 | 1562 | P | E | K | L | L | S | R | K | D | S | 1571 |
| Human CFTR | 1427 | I | Q | K | L | L | N | E | R | S | L | 1436 |

**Figure 5** Alignment of the amino acid sequences of the MRP epitopes for MAbs MRPm6 and MRPrl with comparable regions in several known MRP-related mammalian ABC proteins. (**A**) Epitope sequence of MAb MRPrl. (**B**) Epitope sequence of MAb MRPm6. The sequences of MRP-3, 4, and 5 are not numbered because their complete cDNA sequences have not yet been reported. MRP-6 does not contain a sequence comparable with the MRPrl epitope. Identical amino acids are shaded. Sequences were obtained from the EMBL/GenBank Data Libraries using the following accession numbers: human MRP, L05628; murine mrp, AF022908; human MOAT, U492428; rat moat, L49379; rabbit EBCR, Z49144; human SUR1, L78207; human CFTR, M28668; MRP3, U83659; MRP4, U83660; MRP5, U83661; MRP6, U91318

and peptide GSDLWSLNKE is specific. Similarly, peptide PSDLLQQRGL inhibited the binding of MAb MRPm6 to MRP in a concentration-dependent manner but had no effect on the binding of MAb QCRL-1 at the highest concentration tested (100 μg ml$^{-1}$) (Figure 4B), indicating that the interaction between MAb MRPm6 and peptide PSDLLQQRGL is specific. The locations of the MRPm6, MRPr1 and QCRL-1 epitopes in the MRP molecule are shown in Fig. 4C.

### DISCUSSION

MRP is a relatively newly described multidrug resistance protein that, in mammalian cells, confers a phenotype similar to that associated with overexpression of P-glycoprotein, at least with respect to the classes of drugs to which it confers resistance (Loe et al, 1996*a*; Deeley and Cole, 1997). Consequently, there is considerable interest in determining the relevance of MRP in clinical drug resistance. Several studies examining the expression of MRP in clinical specimens using MRP-reactive MAbs suggest that MRP may be important in a variety of human tumours (Nooter et al, 1995; Filipits et al, 1996*a*, *b*; Flens et al, 1996; Chan et al, 1997). In addition to being essential for studies in clinical samples, MRP-reactive MAbs have also been extremely useful in structure–function analyses and topological studies of MRP (Bakos et al, 1996; Gao et al, 1996; Hipfner et al, 1996, 1997; Loe et al, 1996*b*,*c* and 1997). It may be anticipated that, analogous to MAbs for other ABC proteins (Shapiro and Ling, 1994; O'Riordan et al, 1995; Illing et al, 1997), they will also prove useful for purification and reconstitution studies of MRP. Given their pivotal role in both clinical and experimental investigations of MRP, it is important that these immunoreagents are well characterized.

With the exception of antisera raised against peptide sequences in the relatively highly conserved NBDs of MRP, none of the MRP-reactive antisera or MAbs currently available have been demonstrated to cross-react with P-glycoprotein. This observation is not surprising in view of the very low sequence identity (< 15%) between these two proteins. More recently, however, several ABC transporters with substantially greater similarity to MRP than P-glycoprotein have been cloned and characterized. For example, the amino acid sequence of the multispecific organic anion transporter (MOAT) is approximately 50% identical to that of MRP (Paulusma et al, 1996; Taniguchi et al, 1996), and the recently published partial sequences for human MRP3, MRP4 and MRP5 suggest the existence of mammalian ABC proteins with even greater sequence similarity (Kool et al, 1997). Thus, as the ABC superfamily expands and the number of MRP-related proteins increases correspondingly, the specificity of the MRP-reactive MAbs becomes less certain. Concerns about cross-reactivity can be greatly alleviated by knowing the epitope sequence recognized by a given MAb, which allows direct sequence comparisons. For example, we have previously determined that the sequence of the QCRL-1 epitope is not found in any other ABC protein characterized to date (Hipfner et al, 1996). Indeed, even though the amino acid sequence of murine mrp is 88% identical to that of human MRP, the QCRL-1 heptapeptide epitope is not conserved in the murine sequence, consistent with the lack of cross-reactivity of this MAb with the murine protein (Hipfner et al, 1996; Stride et al, 1996). Thus, knowledge of the QCRL-1 epitope sequence provides assurance that this MAb is a highly specific probe for human MRP and makes it possible to use free peptide to compete for antibody binding in various immunoassays.

An alignment of the amino acid sequences of the MRPr1 and MRPm6 epitopes in human MRP with the comparable regions in several MRP-related mammalian ABC proteins shows that, with the exception of murine mrp, these epitope sequences are poorly conserved (Figure 5). The murine mrp sequence corresponding to the human MRPr1 epitope differs by only one amino acid, a finding consistent with the reported cross-reactivity of MAb MRPr1 with murine mrp (Figure 5A) (Flens et al, 1994; Lorico et al, 1996). In view of the low conservation of the MAb MRPr1 epitope in other known MRP-related proteins, it is reasonable to conclude that this MAb is specific for MRP/mrp. The MRPm6 epitope is also highly conserved in the murine mrp sequence and differs by only two amino acids (Asp$^{1513}$ → Glu$^{1510}$; Leu$^{1520}$ → Ile$^{1517}$) (Figure 5B). These differences are very conservative and, consequently, would not necessarily be expected to alter substantially recognition by MAb MRPm6. Therefore, it is somewhat surprising that this MAb has been reported not to cross-react with murine mrp, a finding we have confirmed by dot-blot analysis of

© Cancer Research Campaign 1998

mrp-enriched membranes (data not shown). These observations, together with the low level conservation of the epitope sequence among other known MRP-related ABC transporters (Figure 5B), indicate that MAb MRPm6 may be considered a human MRP-specific probe at present.

In conclusion, the data shown in the present study together with those of our previous investigations demonstrate that MAbs MRPr1, MRPm6 and QCRL-1 recognize highly specific linear epitopes of MRP and, further, that MAbs MRPm6 and QCRL-1 are specific probes for the human protein. Because each of the three epitopes is in a different region of MRP, these reagents will continue to be extremely valuable tools for both clinical and structure–function analyses of this protein. We have previously described three additional MAbs, namely, MAbs QCRL-2, QCRL-3 and QCRL-4, which detect different conformation-dependent intracellular epitopes in the MRP molecule (Hipfner et al, 1994). We have also determined that all three of these MAbs are capable of inhibiting the transport function of MRP when measured in inside-out membrane vesicles (Loe et al, 1996*b*, *c*, 1997). Accordingly, studies are in progress to map the epitope sequences of these MAbs because they are expected to provide important information about the sites and mechanism(s) of substrate binding and transport by MRP.

### ACKNOWLEDGEMENTS

This investigation was supported by grants from the Medical Research Council of Canada (MT 10519) and the National Cancer Institute of Canada with funds from the Canadian Cancer Society. DR Hipfner is the recipient of a studentship from the Medical Research Council of Canada, RG Deeley is the Stauffer Research Professor of Queen's University and SPC Cole is a Senior Career Scientist of Cancer Care Ontario.

### REFERENCES

Bakos E, Hegedus T, Hollo Z, Welker E, Tusnady GE, Zaman GJR, Flens MJ, Varadi A and Sarkadi B (1996) Membrane topology and glycosylation of the human multidrug resistance-associated protein. *J Biol Chem* **271**: 12322–12326

Barnouin K, Leier I, Jedlitschky G, Pourtier-Manzanedo A, Konig J, Lehmann W-D and Keppler D (1998) Multidrug resistance protein-mediated transport of chlorambucil and melphalan conjugated to glutathione. *Br J Cancer* 77: 201–209

Chan HSL, Lu Y, Grogan TM, Haddad G, Hipfner DR, Cole SPC, Deeley RG, Ling V and Gallie BL (1997) Multidrug resistance protein (MRP) expression in retinoblastoma correlates with rare failure of chemotherapy despite cyclosporine for reversal of P-glycoprotein. *Cancer Res* **57**: 2325–2330

Cole SPC, Bhardwaj G, Gerlach JH, Mackie JE, Grant CE, Almquist KC, Stewart AJ, Kurz EU, Duncan AMV and Deeley RG (1992) Overexpression of a transporter gene in a multidrug-resistant human lung cancer cell line. *Science* **258**: 1650–1654

Deeley RG and Cole SPC (1997) Multidrug resistance in mammalian cells mediated by members of the ATP-binding cassette superfamily: the P-glycoproteins and MRP. In *Molecular Genetics of Drug Resistance*, Hayes J and Wolf CR (eds), pp. 247–298. Harwood Academic Press: The Netherlands

Filipits M, Suchomel RW, Dekan G, Haider K, Valdimarsson G, Depisch D and Pirker R (1996*a*) *MRP* and *MDR1* gene expression in primary breast carcinoma. *Clin Cancer Res* **2**: 1231–1237

Filipits M, Suchomel RW, Zochbauer S, Malayeri R and Pirker R (1996*b*) Clinical relevance of drug resistance genes in malignant diseases. *Leukemia* **10**: S10–S17.

Flens MJ, Izquierdo MA, Scheffer GL, Fritz JM, Meijer CJLM, Scheper RJ and Zaman GJR (1994) Immunochemical detection of the multidrug resistance-associated protein MRP in human multidrug-resistant tumor cells by monoclonal antibodies. *Cancer Res* **54**: 4557–4563

Flens MJ, Zaman GJR, van der Valk P, Izquierdo MA, Schroeijers AB, Scheffer GL, van der Groep P, de Haas M, Meijer CJLM and Scheper RJ (1996) Tissue distribution of the multidrug resistance-associated protein. *Am J Pathol* **148**: 1237–1247

Gao M, Loe DW, Grant CE, Cole SPC and Deeley RG (1996) Reconstitution of ATP-dependent $LTC_4$ transport by co-expression of both half-molecules of human MRP in insect cells. *J Biol Chem* **271**: 27782–27787

Gao M, Yamazaki M, Loe DW, Westlake CJ, Grant CE, Cole SPC and Deeley RG (1998) Multidrug resistance protein: identification of regions required for active transport of leukotriene $C_4$. *J Biol Chem* **273**: 10733–10740

Giaccone G, van Ark-Otte J, Rubio GJ, Gazdar AF, Broxterman HJ, Dingemans A-M, Flens MJ, Scheper RJ and Pinedo HM (1996) MRP is frequently expressed in human lung-cancer cell lines, in non-small-cell lung cancer and in normal lung. *Int J Cancer* **66**: 760–767.

Higgins CF (1992) ABC transporters: from microorganisms to man. *Annu Rev Cell Biol* **8**: 67–113

Hipfner DR, Gauldie SD, Deeley RG and Cole SPC (1994) Detection of the $M_r$ 190,000 multidrug resistance protein, MRP, with monoclonal antibodies. *Cancer Res* **54**: 5788–5792

Hipfner DR, Almquist KC, Stride BD, Deeley RG and Cole SPC (1996) Location of a protease-hypersensitive region in the multidrug resistance protein (MRP) by mapping of the epitope of MRP-specific monoclonal antibody QCRL-1. *Cancer Res* **56**: 3307–3314

Hipfner DR, Almquist KC, Leslie EM, Gerlach JH, Grant CE, Deeley RG and Cole SPC (1997) Membrane topology of the multidrug resistance protein, MRP: a study of glycosylation-site mutants reveals an extracytosolic $NH_2$-terminus. *J Biol Chem* **272**: 23623–23630

Illing M, Molday LL and Molday RS (1997) The 220-kDa rim protein of retinal rod outer segments is a member of the ABC transporter superfamily. *J Biol Chem* **272**: 10303–10310

Jedlitschky G, Leier I, Buchholz U, Barnouin K, Kurz G and Keppler D (1996) Transport of glutathione, glucuronate, and sulfate conjugates by the *MRP* gene-encoded conjugate export pump. *Cancer Res* **56**: 988–994

Kool M, de Haas M, Scheffer GL, Scheper RJ, van Eijk MJT, Juijn JA, Baas F and Borst P (1997) Analysis of expression of *cMOAT (MRP2)*, *MRP3*, *MRP4*, and *MRP5*, homologues of the multidrug resistance-associated protein gene (*MRP1*), in human cancer cell lines. *Cancer Res* **57**: 3537–3547

Leier I, Jedlitschky G, Buchholz U, Cole SPC, Deeley RG and Keppler D (1994) The *MRP* gene encodes an ATP-dependent export pump for leukotriene $C_4$ and structurally related conjugates. *J Biol Chem* **269**: 27807–27810

Loe DW, Deeley RG and Cole SPC (1996*a*) Biology of drug resistance associated with overexpression of the multidrug resistance protein, MRP. *Eur J Cancer* **32A**: 945–957

Loe DW, Almquist KC, Deeley RG and Cole SPC (1996*b*) Multidrug resistance protein (MRP)-mediated transport of leukotriene $C_4$ and chemotherapeutic agents in membrane vesicles: demonstration of glutathione-dependent vincristine transport. *J Biol Chem* **271**: 9675–9682

Loe DW, Almquist KC, Deeley RG and Cole SPC (1996*c*) ATP-dependent 17β-estradiol 17-(β-D-glucuronide) transport by multidrug resistance protein (MRP): inhibition by cholestatic steroids. *J Biol Chem* **271**: 9683–9689

Loe DW, Stewart RK, Massey TE, Deeley RG and Cole SPC (1997) ATP-dependent transport of aflatoxin $B_1$ and its glutathione conjugates by the product of the *MRP* gene. *Mol Pharmacol* **51**: 1034–1041

Lorico A, Rappa G, Flavell RA and Sartorelli AC (1996) Double knockout of the *MRP* gene leads to increased drug sensitivity *in vitro*. *Cancer Res* **56**: 5351–5355

Mirski SEL, Gerlach JH and Cole SPC (1987) Multidrug resistance in a human small cell lung cancer cell line selected in adriamycin. *Cancer Res* **47**: 2594–2598

Muller M, Meijer C, Zaman GJR, Borst P, Scheper RJ, Mulder NH, de Vries EGE and Jansen PLM (1994) Overexpression of the gene encoding the multidrug resistance-associated protein results in increased ATP-dependent glutathione *S*-conjugate transport. *Proc Natl Acad Sci USA* **91**: 13033–13037

Nooter K, Westerman AM, Flens MJ, Zaman GJR, Scheper RJ, van Wingerden KE, Burger H, Oostrum R, Boersma T, Sonneveld P, Gratama JW, Kok T, Eggermont AMM, Bosman FT and Stoter G (1995) Expression of the Multidrug Resistance-associated Protein (*MRP*) gene in human cancers. *Clin Cancer Res* **1**: 1301–1310

Nooter K, Brutel de la Riviere G, Look MP, van Wingerden KE, Henzen-Logmans SC, Scheper RJ, Flens MJ, Klijn JGM, Stoter G and Foekens JA (1997) The prognostic significance of expression of the multidrug resistance-associated protein (MRP) in primary breast cancer. *Br J Cancer* **76**: 486–493

Norris MD, Bordow SB, Marshall GM, Haber PS, Cohn SL and Haber M (1996) Expression of the gene for multidrug-resistance-associated protein and outcome in patients with neuroblastoma. *N Engl J Med* **334**: 231–238

O'Riordan CR, Erickson A, Bear C, Li C, Manavalan P, Wang KX, Marshall J, Scheule RK, McPherson JM, Cheng SH and Smith AE (1995) Purification and characterization of recombinant cystic fibrosis transmembrane conductance regulator from Chinese hamster ovary and insect cells. *J Biol Chem* **270**: 17033–17043

Ota E, Abe Y, Oshika Y, Ozeki Y, Iwasaki M, Inoue H, Yamazaki H, Ueyama Y, Takagi K, Ogata T, Tamaoki N and Nakamura M (1995) Expression of the multidrug resistance-associated protein (*MRP*) gene in non-small-cell lung cancer. *Br J Cancer* **72**: 550–554

Paulusma CC, Bosma PJ, Zaman GJR, Bakker CTM, Otter M, Scheffer GL, Scheper RJ, Borst P and Oude Elferink RPJ (1996) Congenital jaundice in rats with a mutation in a multidrug resistance-associated protein gene. *Science* **271**: 1126–1128

Shapiro A and Ling V (1994) ATPase activity of purified and reconstituted P-glycoprotein from Chinese hamster ovary cells. *J Biol Chem* **269**: 3745–3754

Stride BD, Valdimarsson G, Gerlach JH, Wilson GM, Cole SPC and Deeley RG (1996) Structure and expression of the mRNA encoding the murine multidrug resistance protein (MRP), an ATP-binding cassette transporter. *Mol Pharmacol* **49**: 962–971

Stride BD, Grant CE, Loe DW, Hipfner DR, Cole SPC and Deeley RG (1997) Pharmacological characterization of the murine and human orthologs of multidrug resistance protein (MRP) in transfected human embryonic kidney cells. *Mol Pharmacol* **52**: 344–353

Taniguchi K, Wada M, Kohno K, Nakamura T, Kawabe T, Kawakami M, Kagotani K, Okumura K, Akiyama S and Kuwano M (1996) A human canalicular multispecific organic anion transporter (cMOAT) gene is overexpressed in cisplatin-resistant human cancer cell lines with decreased drug accumulation. *Cancer Res* **56**: 4124–4129

*© Cancer Research Campaign 1998*